# A Treatise on Hernia

**Published:** 1889-07

**Authors:** 


**A Treatise on Hernia. The Radical Cure by the Use of the Buried Antiseptic Animal Suture.** By Henry O. Marcy, A. M., M. D., LL. D., of Boston, Mass., Surgeon to the Private Hospital for Women, Cambridge. President of the Section of Gynecology, Ninth International Congress; late President of the American Academy of Medicine; Member of the British Medical Association; Member of the Massachusetts Medical Society; Fellow Boston Gynecological Society; Corresponding Member of the Medico-Chirurgical Society of Bologna, Italy; Fellow of the American Association of Obstetricians and Gynecologists; late Surgeon U. S. Army, etc., etc. 12mo, pp. vii., 251. Detroit, Mich.: George S. Davis. The Physician's Leisure Library. 1889.

The title of this little book is not a misnomer, for it is in reality a consideration of the whole subject of hernia rather than of its cure by special methods merely. Beginning with the classification of hernias, the author proceeds to the question of causation; the influence of age, sex, occupation, social condition, etc., being taken up in turn and discussed in the usual way. The statistics of Baxter, Malgaigne, and Kingdon have been freely used. The utility of Baxter's tables has been much enhanced by the use of horizontal shaded lines, so that at a glance the relations existing between hernia and locality, age, height, nativity, etc., of 334,321 men examined in the late civil war, can be seen. Then the various anatomical and pathological conditions which act as predisposing causes are discussed at some length.

Chapter I. deals with the formation of the sac, the development of the inguinal canal, congenital, and encysted hernia, while the changes in the hernial sac, the physiological characteristics of the peritoneum and its relations to the hernial sac are most clearly and fully given. In no book or paper on this subject that we can now recall, have we seen more information in an equal space. In fact, we regard this as the best and one of the most important chapters in the book.

The anatomy of the inguinal region is well discussed, but contains nothing more than is usually found in works on the subject.

The author dismisses the subject of trusses rather summarily, giving, however, a clear idea of their mode of action. Like many other surgeons, he finds that cures from their use are very rare and then usually in the young.

The clinical features of irreducible and strangulated hernia are dwelt upon at considerable length, and the description is continually interspersed with valuable hints which the long and ripe experience of Dr. Marcy has suggested. The same is true where operative measures receive consideration. After describing the various means, such as taxis, local applications, etc., adopted for the reduction of hernia, the author then goes at some length into the technique of the open method with buried suture. His rule, "never assume, but determine upon positive knowledge the actual conditions," every surgeon will recognize the value of. Its influence upon Dr. Marcy is apparent throughout the entire work, and we can only hope that more surgeons may memorize it to their own and their patients' advantage. Dr. Marcy strongly advocates operation where other means have failed. He says: "If I write for no other good than to bring the question of operative measures anew under a discriminating judgment, I shall not have labored in vain. When the errors of omission are judged equal to those of commission, then the serious responsibility of the advisor will be viewed in a new but just light." May the time soon come!

The other hernias,—femoral, obturator, ischiatic and umbilical,—are fully considered. The more important points in their symptomatology, diagnosis, and treatment are emphasized, and give the reader an excellent word-picture of them.

The remainder of this book is devoted to the history of the radical cure of hernia from the earliest times. The various expedients adopted up till to-day are described and discussed in a satisfactory way.

Chapter XI. treats of the conditions rendering operative measures advisable, and a plea is made for operation in children not curable by trusses. We think most intelligent surgeons will accept the author's conclusions.

Chapter XII. concludes this little work, and in it the method of operation in the various forms of hernia is described. In inguinal hernia the main indication is to restore the obliquity of the inguinal canal, and this is most thoroughly accomplished, in the author's estimation, by Bassini's method, which in large measure resembles that of Dr. Marcy. In femoral hernia, Macewen's plan is commended, with the reservation, however, that the disposition of the sac in his method is open to more serious objection than in inguinal hernia, inasmuch as it makes a hard, painful swelling, slow to disappear.

The author's method of operation is original, and is based upon what he believes to be a physiological fact, founded upon a long series of studies upon animals undertaken for the purpose, that the aseptically buried animal suture is replaced by a proliferation of connective tissue, which serves as a vital reinforcement of the parts. If true, the use of animal sutures are of value, for this reason, when applied to almost any part of the body; but it will be readily seen how important a service they render in hernia, where such reinforcement is greatly needed. Dr. Marcy's experience in the case of hernia covers a period of eighteen years, and he is undoubtedly the originator of the method ot cure by the use of the buried tendon suture aseptically applied. He was Mr. Lister's first American pupil, and the first, we believe, to adopt and teach antiseptic surgery in the United States. In this he is a rigid disciplinarian, and has devoted a large share of his life to the advancement of modern surgical methods. His list of operations for the cure of hernia now covers about one hundred cases, and, so far as traceable, gives fully ninety per cent. of cures. Dr. Marcy greatly prefers, over all other material for sutures, the tendon from the tail of the kangaroo, which he was the first to use and introduce. This he considers important to apply in a method also his own, his needle being a modification of the Peaslee needle, and by its use the suture is rendered double and continuous. The wound is usually closed by sutures in four layers, the last coapting the skin, but not cutting through it.

So important do we consider the subject and its proper treatment, that we abstract briefly from the author's terse directions. After the now well-known antiseptic preparation of patient, operator, and his surroundings is given—

"(2.) *The Sac.*—Should generally be opened and dissected free, quite within the ring, sutured across at its base, and removed.

"(3.) *The Reconstruction by Buried Animal Sutures.*—The re-formed peritoneum is allowed to retract within the abdominal wall. The pillars of the ring are slightly repressed. The posterior wall of the canal is re-formed by narrowing the internal ring from below, upwards and outwards. This is best, and perhaps easiest, done by buried tendon sutures, applied in an even, continuous, double stitch. Exterior to the cord, the pillars ot the ring are closed downwards, in the same manner, as far as safety to the cord will permit.

"(4.) *The Treatment of the Wound.*—The superficial tissues are brought into juxtaposition by a line of continuous, buried, animal sutures, and the skin coapted by a running, blind stitch, taken from side to side, through the deep layer of the skin. In this way all of the divided tissues are rejoined, and drainage is not required. The line of the incision is dried, dusted with iodoform, and sealed with iodoform collodion. A soft wool pad is usually applied for protection, but is required for no other purpose."

The author frequently emphasizes his belief that primary union, without pain or swelling, will always follow the conjunction of aseptic vitalized tissues.

Dr. Marcy's admirable book, like works of its size, must be judged as a whole, and examined in this way we do not doubt its readers will put it down with a feeling of satisfaction. It is a terse, meaty consideration of hernia, and well deserves a place in the surgeon's library.

The type and wood-cuts are excellent when one considers the price (twenty-five cents) of the book. With such books as this, the success of the Physician's Leisure Library is assured.

